# An Intelligent Epileptic Prediction System Based on Synchrosqueezed Wavelet Transform and Multi-Level Feature CNN for Smart Healthcare IoT

**DOI:** 10.3390/s22176458

**Published:** 2022-08-27

**Authors:** Kunpeng Song, Jiajia Fang, Lei Zhang, Fangni Chen, Jian Wan, Neal Xiong

**Affiliations:** 1 School of Information and Electronic Engineering, Zhejiang University of Science and Technology, Hangzhou 310023, China; 2 Department of Neurology, The Fourth Affiliated Hospital, Zhejiang University School of Medicine, Yiwu 322000, China; 3 Department of Computer Science and Mathematics, Sul Ross State University, Alpine, TX 79830, USA

**Keywords:** seizure prediction, Internet of Things, convolutional neural network, synchrosqueezed wavelet transform, electroencephalogram (EEG)

## Abstract

Epilepsy is a common neurological disease worldwide, characterized by recurrent seizures. There is currently no cure for epilepsy. However, seizures can be controlled by drugs and surgeries in about 70% of epileptic patients. A timely and accurate prediction of seizures can prevent injuries during seizures and improve the patients’ quality of life. In this paper, we proposed an intelligent epileptic prediction system based on Synchrosqueezed Wavelet Transform (SWT) and Multi-Level Feature Convolutional Neural Network (MLF-CNN) for smart healthcare IoT network. In this system, we used SWT to map EEG signals to the frequency domain, which was able to measure the energy changes in EEG signals caused by seizures within a well-defined Time-Frequency (TF) plane. MLF-CNN was then applied to extract multi-level features from the processed EEG signals and classify the different seizure segments. The performance of our proposed system was evaluated with the publicly available CHB-MIT dataset and our private ZJU4H dataset. The system achieved an accuracy of 96.99% and 94.25%, a sensitivity of 96.48% and 97.76%, a specificity of 97.46% and 94.07% and a false prediction rate (FPR/h) of 0.031 and 0.049 FPR/h on the CHB-MIT dataset and the ZJU4H dataset, respectively.

## 1. Introduction

Epilepsy is caused by the abnormal electrical activities of neurons in the brain, leading to convulsions and loss of consciousness. According to the report published by the World Health Organization (WHO), epilepsy has become one of the most common neurological diseases globally, affecting about 50 million people worldwide [1]. Epileptic patients are three times more likely to die prematurely because of the accidental injuries or the brain damage caused by continuous seizures when compared with the general population. Therefore, having an effective framework to predict epileptic seizures is significant for epileptic patients.

In recent years, Internet of Things (IoT) technology has been flourishing and made remarkable achievements in smart home [2,3], intelligent logistics [4] and intelligent transportation [5]. At the same time, smart healthcare is deeply integrated with the IoT, artificial intelligence and the medical industry [6,7], providing a new framework for hospitals, doctors and patients. Telemedicine and the diagnosis of chronic diseases with wearable devices, such as epilepsy, diabetes and heart disease, are smart healthcare. Doctors can monitor patients’ health status in real time through IoT technology and wearable devices. Academia is also committed to researching and explore IoT solutions for seizure prediction [8,9]. The development of wireless technologies has ensured low latency [10,11] and power efficiency [12,13] for IoT networks. Artificial intelligence techniques can classify the EEG signals recorded in real time to achieve effective prediction of seizures.

In this study, a latest seizure prediction framework is suggested based on the IoT network and deep learning. We proposed a novel Synchrosqueezed Wavelet Transform (SWT) and Multi-Level Feature Convolutional Neural Network (MLF-CNN) system to predict seizure onset from Electroencephalogram (EEG) signals. EEG measures voltage fluctuations generated between neurons in the brain, which is the most common signal for monitoring brain state and widely used in the diagnosis of epilepsy. The SWT was used to measure the energy changes in EEG signals caused by seizures within a well-defined TF plane. The MLF-CNN model was used to extract multi-level features from the processed EEG signals and classify the different seizure segments. IoT technology enabled real-time monitoring, transmission, and recording of EEG signals from epileptic patients, and it provided effective seizure prediction together with deep learning as previously mentioned.

Currently, the prediction of epilepsy mainly focuses on measuring changes of the EEG signals before a seizure onset, known as the preictal. A lot of research has been carried out using the artificial intelligence approach for the prediction and classification of epileptic seizures. Therefore, many artificial intelligence algorithms have been developed, which can be divided into two categories: machine learning and deep learning. These algorithms are generally based on advanced signal processing techniques and feature extraction schemes.

Traditional machine learning methods require manual feature extraction, which significantly increases labor costs and human errors. Deep learning has excellent applications in various fields. For example, the AlphaGo from Deep Mind is based on a variety of deep neural network technology [14]. Therefore, deep learning methods for predicting epileptic seizures are gaining more and more attention. One of the characteristics of deep learning is that relevant features can be extracted automatically using a network model. Zhou et al. [15] used the Fast Fourier Transform (FFT) and Convolutional Neural Network (CNN) model to predict the onset of epilepsy. Their proposed model achieved an accuracy of 97.7% on the Freiburg dataset and 91.1% on the CHB-MIT dataset. They also concluded that the performance of the frequency domain is better than that of the time domain. Shahbazi et al. [16] applied Short-Time Fourier Transform (STFT) to the EEG signals to construct a multichannel EEG image. A three-layer CNN was also used to extract spatial features from STFT images. Additionally, a Long-Short Time Memory network (LSTM) and a post-processing procedure were utilized to automatically extract temporal features from the EEG signal, reducing the impact of individual prediction errors. This algorithm achieved a sensitivity of 98.21% and a false prediction rate of 0.13 FPR/h on the CHB-MIT dataset. Truong et al. [17] applied the STFT algorithm to extract time and frequency domain information from a 30-s EEG window. The extracted information was used to train a three-layer CNN network, which achieved a sensitivity of 81.4%, 81.2% and 75% on the Freiburg Hospital intracranial EEG dataset, CHB-MIT dataset and the American Epilepsy Society Seizure Prediction Challenge dataset, respectively. In addition, Liu et al. [18] used both frequency and time domains to develop their prediction algorithm. The time-domain data were processed using principal component analysis (PCA). The frequency-domain data were obtained using FFT. The final algorithm was tested on two cases obtained from the CHB-MIT dataset, and an area under the curve (AUCs) of 0.85.

The wavelet transform method is another method commonly used for time–frequency analysis. Various methods have been proposed to transform the EEG to predict the onset of epileptic seizures. Khan et al. [19] used a seven-layer CNN to predict the onset of epilepsy. This method achieved a sensitivity of 87.8% and a false prediction rate of 0.142 FP/h. Hussein [20] used the Continuous Wavelet Transform (CWT) to transform the EEG signal into an image-like format. A Semi-Dilated Convolutional Network (SDCN) was then used to expand the receiver in time domain while maintaining the same resolution within the frequency domain. Aliyu et al. [21] used Discrete Wavelet Transform (DWT) to remove noise and selected the most relevant prediction features from 20 extracted features. The features that were significantly correlated with the onset of epilepsy were identified and used to train a LSTM model. This method significantly reduced the parameters required to train the model and improved the model’s performance. Shoeibi et al. [22] used tunable-Q wavelet transform (TQWT) to decompose the EEG signals into different sub-bands, and 15 different fuzzy entropies were extracted from 9 sub-bands of TQWT. By an autoencoder and breeding swarm optimization (ANFIS-BS) method, they obtained an fantastic accuracy of 99.74% in classifying into two classes on the Bonn dataset.

Other studies [23,24,25,26,27] used the raw EEG signals without any preprocessing and also achieved good results in predicting epileptic seizures. Besides, Cao and Hu et al. [28,29,30,31] achieved multi level prediction of epilepsy and obtained good results using the Mean Amplitude Spectrum (MAS). Ozdemir [32] proposed a novel method based on Fourier-based Synchrosqueezing Transform, to optimize a ResNet50 network, and also achieved good results in both epilepsy detection and prediction.

The two main factors affecting the prediction accuracy of current algorithms were effective signal processing methods and the development of an efficient deep learning model. Conventional Fourier transform and wavelet transform have a limited resolution as they are constrained by the uncertainty principle. According to this principle, the frequency resolution decreases when the temporal resolution increases and, conversely, the temporal resolution decreases when the frequency resolution increases. Therefore, to resolve these issues, SWT-based CNN prediction model is introduced and integrated into a smart IoT network framework for epileptic seizure. The contributions of this paper are as follows.

(1) A smart epileptic seizure prediction IoT framework using deep learning technology was proposed. It can provide services for hospitals, doctors and patients. For epileptic patients who cannot be operated on, it can send warnings before epileptic seizures, reduce psychological pressures and improve their quality of life.
(2) Synchrosqueezed Wavelet Transform (SWT) was introduced to process and analyze EEG signals. SWT is a signal rearrangement algorithm based on wavelet transform in the time–frequency domain. It can clearly represent the sudden energy discharges and provide highly localized time–frequency energy distributions of EEG signals, which can help to improve the classification accuracy.
(3) A novel Multi-Level Feature Convolutional Neural Network (MLF-CNN) was established to extract features from different dimensions automatically. The extracted features were concatenated and sent to a hierarchical neural network which consisted of 2 fully-connected layers, 2 dropout layers followed by a softmax layer developed for EEG feature learning and epileptic states classification. The proposed MLF-CNN achieved better performance compared with some other research, which was demonstrated in Section 4.
(4) The proposed epileptic seizure prediction system was validated not only on the public CHB-MIT dataset but also on the private ZJU4H dataset collected by our cooperation hospital. Great performance was achieved by both of them. This demonstrated that our approach could provide robust epilepsy prediction.

The remainder of this paper is organized as follows. Section 2 describes the entire experimental process, including the datasets used for the study, development of the SWT algorithm and data preprocessing. Section 3 discusses the development of the MLF-CNN prediction model. Section 4 describes the performance of our proposed model using two datasets, which we collected ZJU4H dataset at hospital. Finally, in Section 5, the research findings are summarized and recommendations for further research are given.

## 2. Materials and Methods

### 2.1. The Smart Epileptic Seizure Prediction IoT Framework

**Figure 1:**
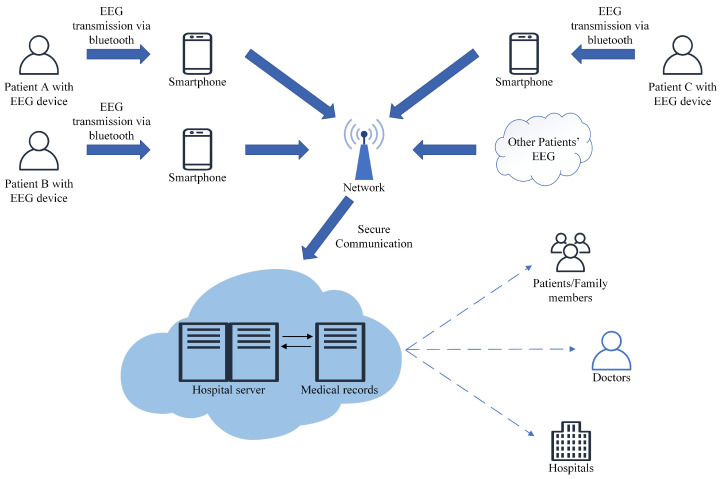
The smart IoT network framework for epileptic seizure prediction.

The proposed smart epileptic seizure prediction IoT framework is shown in Figure 1. First, the EEG data of epileptic patients are collected and transmitted to the patients’ mobile phones by portable EEG devices via Bluetooth. Second, the smartphones upload EEG data to the hospital server through secure mobile communication network. Chaos-based communication systems have made great progress. They can provide a high level of privacy in data transmission [33,34]. The server saves the EEG data and associates it with the patients’ medical records before processing it. Then the server will execute the SWT based MLF-CNN system to predict epileptic seizures. Finally, if the onset is predicted, an alert will be sent to the doctors and the patients (or family members). They can take prompt measures to prevent serious consequences. Moreover, after obtaining the consent of patients, the hospital can utilize these data to conduct in-depth research on epilepsy diagnosis.

### 2.2. Segmentation of EEG Signal

**Figure 2:**
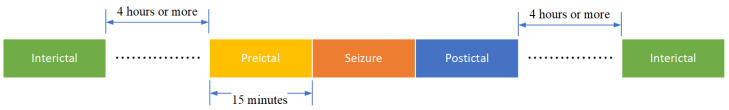
All stages of epileptic EEG signals.

The EEG signal was divided into several sections, as shown in Figure 2. Generally, epilepsy signals consist of four parts: interictal, preictal, ictal, and postictal. At present, there is no uniform standard for the partition of interictal and preictal periods. Shahbazi, Truong, Khan, et al. [16,17,19] defined preictal as being 30 min before seizure onset, and Hussein, Daoud, Dissanayake, et al. [20,26,35] defined it as one hour. A long preictal EEG time increases the stress and anxiety for patients, whereas a short preictal data may not provide enough medical information to intervene appropriately. Therefore, a preictal time of 15 min was deemed the most appropriate, as shown in Figure 2. Moreover, interictal data was obtained at a minimum of 4 h before seizure onset and 4 h after the end of the seizure. This data was acquired to reduce the impact of noise on the model’s prediction accuracy.

### 2.3. Data Acquisition

Epilepsy can be seen in all age groups. However, the incidence rate of children’s epilepsy is higher than that of adults. In total, 80% of epilepsy patients had their first seizure before they were 18 years old [36]. Therefore, the prevention and treatment of children’s epilepsy is particularly important. The CHB-MIT dataset [37] and ZJU4H dataset were used to train and validate the deep learning model. The characteristics of the patients obtained from the CHB-MIT dataset and ZJU4H dataset are summarized in Table 1 and Table 2, respectively. As we can see, patients in both datasets are young. All EEG signals were acquired using the standard International 10–20 EEG electrode system. The CHB-MIT dataset is currently the most widely used publicly available dataset in the field of epilepsy. The dataset was collected at Boston Children’s Hospital and consisted of 24 EEG recordings of intractable epilepsy [37]. Signals from all cases were sampled at a rate of 256 Hz and a resolution of 16. The dataset also provided the start and end times of each epileptic seizure for each patient. Since the time information of subject 24 was missing, only the EEG data of the first 23 subjects were included in this study.

The ZJU4H dataset was collected from the Fourth Affiliated Hospital Zhejiang University School of Medicine, including 8 epileptic patients’ resting-state scalp EEG data. The EEG data were recorded with Nicolet V32 equipment at a sampling rate of 1000 Hz. The duration of the EEG recording ranged between 2 to 12 h. The seizure onset and offset time intervals were manually annotated by clinical experts after a visual inspection.

### 2.4. Synchrosqueezed Wavelet Transform

The Synchrosqueezed Wavelet Transform (SWT) is a signal rearrangement algorithm in the time–frequency domain based on wavelet transform. This algorithm can depict the characteristics of a time–frequency spectrum better than Continuous Wavelet Transform (CWT). Meanwhile, studies have shown that the Synchrosqueezed Fourier Transform (FSST) can improve the classification of physiological signals [32,38]. However, STFT and FSST have fixed and single time–frequency resolutions. CWT and SWT have good frequency resolution at low frequency and time resolution at high frequency. Therefore, time–frequency spectrum rearrangement was used to increase the TF concentration and resolution [39,40,41]. To our knowledge, this is the first application of SWT for seizure prediction using EEG data. The SWT is developed using the following formula.

For a given signal $x\left(t\right)\in L^{2}\left(R\right)$, the CWT under the continuous wavelet ψ(t), the time and frequency domain are defined as described in Equations (1) and (2), respectively.

(1) $$W_{x}\left(a,b\right)=\langle x,\psi_{a,b}\rangle =\int_{-\infty }^{\infty }x\left(t\right)\frac{1}{a}\bar{\psi \left(\frac{t-b}{a}\right)}dt$$

In Equation (1), $\psi_{a,b}\left(t\right)=\frac{1}{a}\psi \left(\frac{t-b}{a}\right)$, *a* is the scale factor, and *b* is the translation factor.

(2) $$W_{x}\left(a,b\right)=\langle \hat{x},{\hat{\psi }}_{a,b}\rangle =\int_{-\infty }^{\infty }\hat{x}\left(\xi \right)\bar{\hat{\psi }\left(a\xi \right)}e^{i2\pi b\xi }d\xi$$

A higher time–frequency resolution of the signal is then achieved by transforming scale variables to frequency variables using reassigns. For a general signal x(t), the phase transformation $\omega_{x}\left(a,b\right)$ is defined as:

(3) $$\omega_{x}\left(a,b\right)=\frac{\partial }{\partial b}W_{x}\left(a,b\right)/i2\pi W_{x}\left(a,b\right)$$

where by $W_{x}\left(a,b\right)\neq 0$. Then the SWT in the time–frequency plane is expressed as:

(4) $$T_{x}\left(\xi ,b\right)=\int_{R}W_{x}\left(a,b\right)\delta \left(\omega_{x}\left(a,b\right)-\xi \right)\frac{da}{a}$$

**Figure 3:**
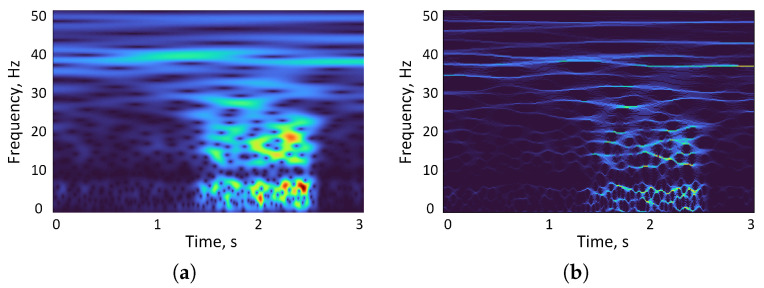
The comparison of CWT and SWT: (**a**) CWT example, (**b**) SWT example.

The time–frequency representations of the proposed SWT method and the traditional CWT time–frequency analysis method are compared in Figure 3. As expected in the preictal and interictal EEG segment, SWT has a higher TF resolution when compared with CWT. In Figure 3a, the change of energy after CWT is ambiguous and gentle. However, the change of energy after SWT as shown in Figure 3b is vivid and sharp, which can strongly reflect the EEG changes before seizures. In this paper, the proposed deep learning network model was based on EEG signals which was processed by SWT and used as input data.

## 3. Our Proposed MLF-CNN Scheme

### 3.1. Preprocessing of the EEG Signals

The CHB-MIT dataset contains long periods of EEG signals which are difficult to process. Therefore, the time-domain data were divided into different segments to overcome this problem. Since the interictal period was much longer than the preictal period, sections of the interictal data were randomly selected to balance the data between the two phases and achieve a ratio of 1:1. According to previous work and clinical recommendations [42], the epileptic EEG signal is mainly concentrated at 0-50 Hz. Therefore, only signal frequencies between 0 and 50 Hz were used. Since the dataset had missing channels, 18 of the 23 channels shared in the entire dataset were selected, including FP1-F7, F7-T7, T7-P7, P7-O1, FP1-F3, F3-C3, C3-P3, P3-O1, FP2-F4, F4-C4, C4-P4, P4-O2, FP2-F8 F8-T8, T8-P8, P8-O2, FZ-CZ, CZ-PZ. After segmentation and SWT processing for each channel, the processed data were resized to 128 × 128 × 18 using bilinear interpolation to fit the neural network better and reduce the network’s training cost. The 1 s and 3 s EEG segments were evaluated in this study.

For the ZJU4H dataset, 16 channels were selected based on the recommendation of a neurologist from the Fourth Affiliated Hospital Zhejiang University School of Medicine. These channels included Fp1-Ref, Fp2-Ref, F3-Ref, F4-Ref, C3-Ref, C4-Ref, P3-Ref, P4-Ref, O1-Ref, O2-Ref, F7-Ref, F8-Ref, T3-Ref, T4-Ref, T5-Ref, T6-Ref. The rest of processing steps are the same as the CHB-MIT dataset.

### 3.2. CNN Architecture

The CNN architecture was used in our approach. The VGG16 neural network is a deep CNN developed by the Computer Vision Group at the University of Oxford, which was designed to explore the relationship between depth and performance of CNN. The VGG16 structure is simple, consisting of 13 convolutional layers and 3 fully connected layers. Each convolutional layers makes use of 3×3 convolutional kernels with 1 stride. The max-pooling layers are also utilized.

**Figure 4:**
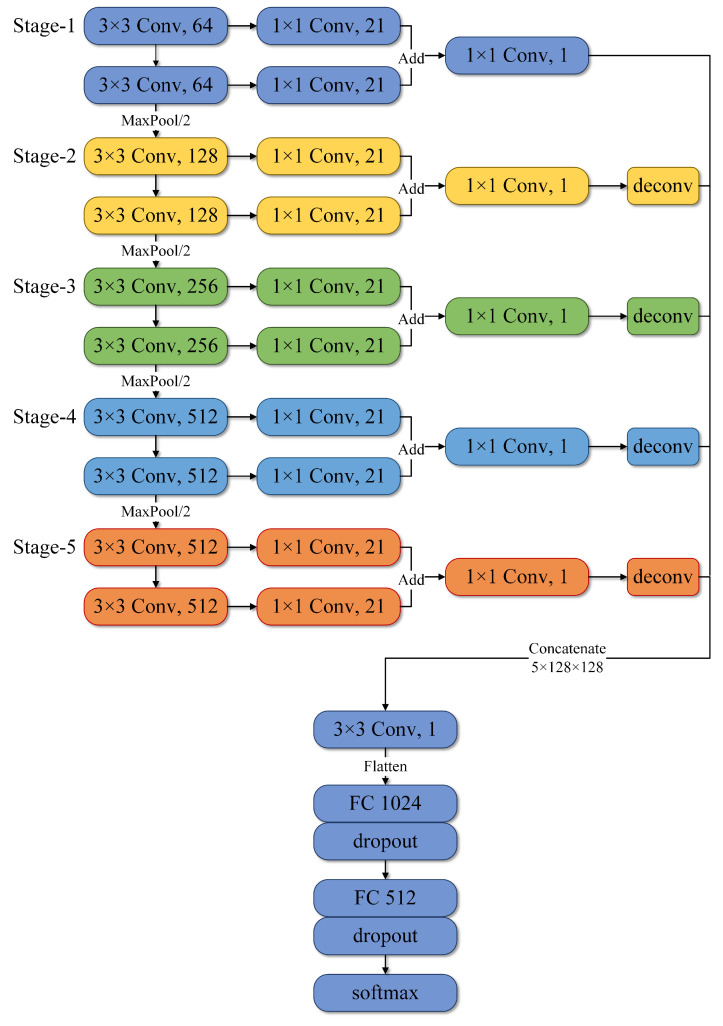
The proposed MLF-CNN architecture.

However, VGG16 was originally developed to process Red, Green, and Blue (RGB) images at 224×224. This model structure was used to classify conventional images, but not suitable for evaluating the time–frequency image obtained following SWT processing. Therefore, we developed a new MLF-CNN model based on VGG16 as follows. We first limited the number of convolution layers in the fourth and fifth blocks to the top-level receptive field. We then used a 1×1 convolution kernel to process the results of each convolution layer into vectors of the same dimensional size. Then the vectors obtained in each block were combined and deconvoluted to enlarge the feature size. After this step, we obtained five same-size feature maps. Finally, the five enlarged feature maps were concatenated together and classified using softmax. The detailed network structure is shown in Figure 4. The five feature maps were obtained from five different blocks, with each feature map containing information at different dimensions. The feature combination with different dimensions reduced the defects of the VGG16 network, improved the network performance, and resolved the instability phenomenon within the metrics.

## 4. Performance Analysis

The algorithm presented in this study was based on the time–frequency analysis method illustrated in Section 2. All training and testing procedures were executed on an RTX TITAN graphics processing unit with 32G random access memory using Python 3.6, Keras 2.3.0, and TensorFlow 2.2.0.

Two EEG segment lengths and two models were used for training and testing. The different lengths were used to investigate the effect of the signal segment size on the experimental results. The performance of our model in predicting the onset of epilepsy was compared with CWT. The evaluation metrics used in this paper were accuracy, sensitivity, and specificity (also called recall). Sensitivity is the percentage of true positive samples in the actual positive samples, and specificity is the percentage of true negative samples in the actual negative samples. If the sensitivity of the proposed model is too low, the rate of false negatives will increase. False negatives may result in a delay in the provision of treatment. Conversely, if specificity is poor, the false positive rate will increase. False positives could lead to unnecessary treatment interventions and increase anxiety levels amongst patients. Therefore, the epilepsy algorithm needs to have a high sensitivity and specificity to be feasible for clinical use.

**Figure 5:**
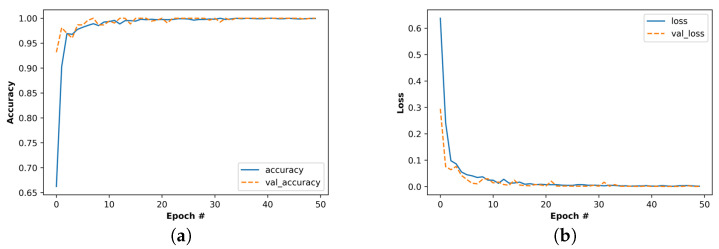
Training performance of the 3 s-segment time–frequency map, (**a**) training and validation accuracies, (**b**) cross entropy loss.

All the 3 s segments were trained using 5-fold cross-validation to reduce the risk of overfitting the model. Figure 5. shows the accuracy and loss changes during the training of 3 s SWT segments. It is important to note that the accuracy values converge at the upper limit, and the loss values converge at the lower limit. Therefore, no overfitting or underfitting is observed in any of the evaluated models. Although the SWT-3s model converged before the 50 epochs, the epoch was still fixed at 50 to control the variables and maintain the consistency of the experimental conditions.

Table 3 shows the training results based on the VGG16 model with CWT and SWT time–frequency images, which were obtained from each patient. For a 1 s-duration segment, the highest prediction performance was achieved by both the CWT images and SWT images under the VGG16 model for patient chb08. However, large variations in the prediction performance of both the CWT and SWT images were noted. For example, in the CWT image for patient chb04, the accuracy and specificity were less than 60.00%, but the sensitivity reached 93.52%. Similarly, for the SWT image of patient chb10, the accuracy, sensitivity, and specificity were 68.20%, 72.24%, and 65.41%, respectively. The large variations in the algorithm’s prediction accuracy for epilepsy in different patients could increase the rate of false predictions leading to late or inappropriate interventions by healthcare professionals.

To compare the effect of different segment lengths on the experimental results, we repeated the above experiments using 3 s-duration segments, and the detailed results are shown in Table 4. Since the 3 s segments contained more information than the 1 s segment, the overall average performances of both CWT and SWT improved. The average accuracy, sensitivity, and specificity for CWT were 93.84%, 93.47%, and 94.21%, respectively. Similarly, the average accuracy, sensitivity, and specificity for SWT were 94.99%, 94.50%, and 95.25%, respectively. The CWT algorithm achieved very high sensitivity and specificity for most patients, and eventually, four patients (chb01, chb11, chb19, chb20) achieved a 100% result. However, the model still performed poorly in some patients, such as in patient chb02 with a specificity of 63.92% and patient chb04 with a sensitivity of 50.00%. Similarly, although the overall performance of SWT was improved compared with CWT, the specificity of patient chb16 and the sensitivity of patient chb21 were still less than satisfactory.

To address the problem of reasonable accuracy but poor sensitivity and specificity in some patients, we proposed the MLF-CNN model, which is detailed in Section 3. Since, overall, the performance of SWT was better than that of CWT, only the 1 s and 3 s SWT segments were integrated into the MLF-CNN model for further testing. The test results are shown in Table 5. The performance of the MLF-CNN model improved compared with VGG16 and eventually achieved an overall accuracy, sensitivity, and specificity of 96.99%, 96.48%, and 97.46%. Furthermore, the model provided more stable sensitivity and specificity results and achieved a false prediction rate of 0.031 FPR/h. For the 1 s-duration SWT, the lowest sensitivity was 78.75% (chb06) and the lowest specificity was 74.17% (chb10). Moreover, the results of all metrics were improved with the 3 s-duration SWT. The lowest prediction accuracy for the 3 s-duration SWT was 85.00% (chb14). The difference in sensitivity and specificity was less than 3%.

**Figure 6:**
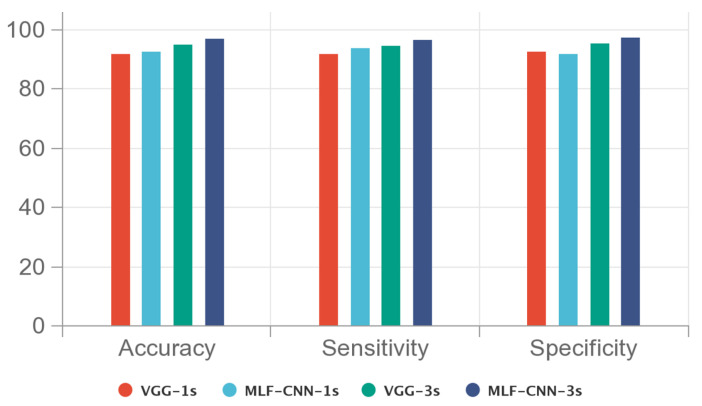
The comparison of results under different segments and models using CHB-MIT dataset and SWT time–frequency images.

**Figure 7:**
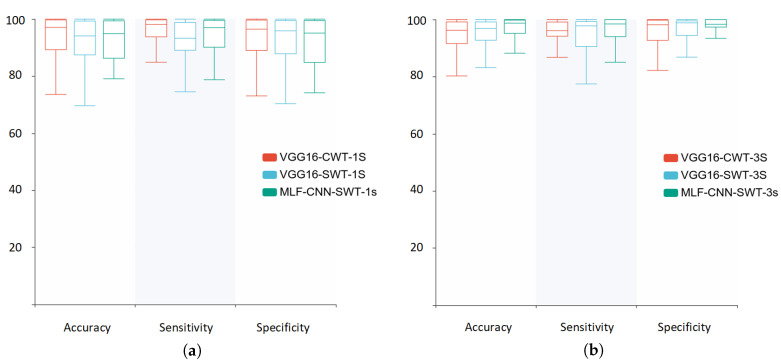
The distribution of experimental results. (**a**) 1 s segments, (**b**) 3 s segments.

The results of the above experiments using SWT were integrated and plotted as a graph shown in Figure 6. The findings indicate that the 3 s segment provided more information and effectively improved the prediction performance when compared with the 1 s segment. The proposed MLF-CNN prediction algorithm further improved the accuracy, sensitivity, and specificity of the time–frequency classification of epilepsy on the EEG signal. Furthermore, the prediction results were more stable, as shown in Figure 7.

In addition, we used the ZJU4H dataset to validate the MLF-CNN model. The experiment results are shown in Table 6. However, only the 1 s segments were used for validation due to the limited data was available. The model’s overall accuracy, sensitivity, and specificity following validation were 94.25%, 97.76%, and 94.07%, respectively. It also achieved a false prediction rate of 0.049 FPR/h. This finding shows that our method can perform well on other datasets.

The literature findings evaluating the accuracy of classification-based seizure prediction models on the CHB-MIT dataset are summarized in Table 7. Since the seizure prediction has already achieved high accuracy in recently published studies, we focused on sensitivity and specificity. A time–frequency map model using the discrete wavelet transform as input to a five-layer CNN [19] achieved an average sensitivity of 87.8% and specificity of 85.8% on the CHB-MIT dataset. Truong proposed method achieved a sensitivity and specificity of less than 85% [17]. Compared with previous works, our proposed method achieved the highest accuracy (96.99%), sensitivity (96.48%), and specificity (97.46%) on the CHB-MIT scalp dataset.

## 5. Conclusions and the Future Work

This paper proposed an intelligent epileptic prediction system based on Synchrosqueezed Wavelet Transform (SWT) and Multi-Level Feature Convolutional Neural Network (MLF-CNN) for Smart healthcare IoT network, in which the EEG data of epileptic patients are collected, transmitted and processed to provide prompt seizure onset alert for doctors and patients to take necessary measures. In the SWT based MLF-CNN model, time–frequency images of EEG segments were obtained using SWT, which provides a higher TF resolution of EEG signals. Then, the processed time–frequency images were used to train MLF-CNN model to extract multi-level feature information. The proposed system was tested on the CHB-MIT dataset and the ZJU4H dataset. To the best of our knowledge, this is the first study that used SWT to analyze epileptic EEG signals and to classify TF images using a CNN model. Our model achieved a high level of accuracy on both CHB-MIT and ZJU4H datasets. Furthermore, our model also achieved higher sensitivity and specificity when compared with other existing epilepsy prediction models.

Although the proposed method performed well, the sample size was relatively, small which probably limits the generalizability of the research findings. For the future work, novel signal processing techniques will be developed to characterize the EEG signals more effectively based on the premise of collecting more EEG data. Moreover, individual differences in the EEG signals among different patients are not well studied. More researches should evaluate these variations to further enhance the generalizability of the model. At last, it is worth noting that we adopted the CHB-MIT dataset and ZJU4H dataset, which mainly collected the data of young patients. Since most patients have their first onset in childhood, the prevention and treatment of children’s epilepsy is particularly important. Significantly, the status of adult and elderly patients should also be reviewed, and we will consider them in the future work.

## Figures and Tables

**Table 1:** Illustrations of the CHB-MIT dataset we used.

Patient	Gender	Age	The Preictal We Used/min
chb01	F	11	105
chb02	M	11	45
chb03	F	14	105
chb04	M	22	60
chb05	F	7	75
chb06	F	2	135
chb07	F	15	45
chb08	M	4	75
chb09	F	10	60
chb10	M	3	105
chb11	F	12	45
chb12	F	2	585
chb13	F	3	180
chb14	F	9	120
chb15	M	16	300
chb16	F	7	150
chb17	F	12	45
chb18	F	18	90
chb19	F	19	45
chb20	F	6	120
chb21	F	13	60
chb22	F	9	45
chb23	F	6	105

**Table 2:** Illustrations of the ZJU4H dataset we used.

Patient	Gender	Age	The Preictal We Used/min
pa01	M	14	45
pa02	M	4	45
pa03	F	1	15
pa04	M	11	75
pa05	M	13	30
pa06	M	9	75
pa07	M	1	15
pa08	M	12	15

**Table 3:** The comparison of 1 s CWT and SWT segments under VGG16 model.

Patient	CWT-1s			SWT-1s		
	Accuracy	Sensitivity	Specificity	Accuracy	Sensitivity	Specificity
chb01	99.88	99.90	99.85	99.90	99.95	99.85
chb02	92.71	95.56	90.20	93.97	93.11	94.80
chb03	99.77	99.72	99.83	99.86	99.71	100.00
chb04	59.60	93.52	55.39	98.20	98.84	97.52
chb05	98.61	99.58	97.68	88.47	92.10	85.42
chb06	80.50	82.43	78.78	82.41	84.39	80.65
chb07	96.32	97.57	95.13	86.45	85.70	87.23
chb08	100.00	100.00	100.00	100.00	100.00	100.00
chb09	98.93	99.73	98.15	97.50	96.90	98.14
chb10	77.16	74.38	80.66	68.20	72.24	65.41
chb11	100.00	100.00	100.00	99.27	98.56	100.00
chb12	97.20	97.84	96.48	99.50	99.81	99.17
chb13	88.68	98.69	82.08	94.31	94.43	94.20
chb14	93.13	91.32	95.11	94.10	93.41	94.83
chb15	89.86	94.06	86.39	84.42	88.64	81.03
chb16	78.21	70.80	93.81	77.47	70.00	93.83
chb17	99.74	100.00	99.48	93.82	89.55	99.12
chb18	97.55	98.46	96.67	94.80	93.30	96.41
chb19	99.69	99.38	100.00	99.38	98.77	100.00
chb20	100.00	100.00	100.00	99.89	99.83	99.94
chb21	83.39	79.87	87.85	83.04	82.34	83.76
chb22	97.11	98.12	96.13	89.08	89.71	88.47
chb23	96.79	95.80	97.81	93.25	90.90	95.90
Average	92.50	94.29	92.66	92.01	91.83	92.86

**Table 4:** The comparison of 3 s CWT and SWT segments under VGG16 model, using 5-fold cross validation.

Patient	CWT-3s			SWT-3s		
	Accuracy	Sensitivity	Specificity	Accuracy	Sensitivity	Specificity
chb01	100.00	100.00	100.00	100.00	100.00	100.00
chb02	82.00	99.03	63.92	90.50	90.29	90.72
chb03	97.60	96.09	99.54	91.80	86.48	98.63
chb04	70.56	50.00	90.22	100.00	100.00	100.00
chb05	95.68	96.12	95.30	96.59	96.12	97.01
chb06	88.60	84.88	92.57	90.70	85.55	96.16
chb07	95.83	98.33	93.33	99.17	99.17	99.17
chb08	99.17	98.30	100.00	99.72	99.43	100.00
chb09	99.72	99.43	100.00	98.89	98.86	98.91
chb10	93.13	95.20	91.26	93.75	96.05	91.53
chb11	100.00	100.00	100.00	99.17	98.36	100.00
chb12	99.17	98.77	99.54	98.33	97.79	98.85
chb13	95.83	92.09	99.45	94.09	88.70	99.18
chb14	83.45	74.50	92.91	87.41	84.23	90.78
chb15	96.38	93.75	99.00	96.88	97.75	96.00
chb16	90.21	94.62	85.00	79.38	96.15	59.55
chb17	93.00	90.48	97.30	95.00	100.00	86.49
chb18	98.75	99.23	98.18	99.17	98.85	99.55
chb19	100.00	100.00	100.00	100.00	100.00	100.00
chb20	100.00	100.00	100.00	99.80	100.00	99.54
chb21	85.56	95.45	76.09	81.67	69.89	92.93
chb22	96.25	97.50	95.00	93.75	90.83	96.67
chb23	97.27	96.12	98.29	99.09	99.03	99.15
Average	93.84	93.47	94.21	94.99	94.50	95.25

**Table 5:** The performance of 1 s and 3 s SWT segments under the proposed MLF-CNN model.

Patient	SWT-1s			SWT-3s		
	Accuracy	Sensitivity	Specificity	Accuracy	Sensitivity	Specificity
chb01	99.90	99.90	99.90	100.00	100.00	100.00
chb02	94.58	98.05	91.58	97.00	97.09	96.91
chb03	99.94	100.00	99.89	99.60	99.29	100.00
chb04	98.44	97.46	99.45	100.00	100.00	100.00
chb05	84.58	86.19	83.11	95.68	93.69	97.44
chb06	80.74	78.75	83.04	93.37	90.07	96.88
chb07	90.13	94.59	86.48	98.75	99.17	98.33
chb08	99.29	100.00	98.59	100.00	100.00	100.00
chb09	96.88	98.79	95.10	98.89	98.86	98.91
chb10	79.10	86.56	74.17	93.75	94.35	93.17
chb11	100.00	100.00	100.00	100.00	100.00	100.00
chb12	98.50	99.12	97.90	99.17	100.00	98.38
chb13	93.75	92.43	95.15	98.06	98.02	98.09
chb14	83.54	87.86	80.10	84.83	82.89	86.88
chb15	88.02	92.69	84.27	95.34	93.42	97.46
chb16	83.68	82.14	85.38	92.71	95.38	89.55
chb17	99.47	99.21	99.74	99.00	100.00	97.30
chb18	90.19	95.72	85.86	98.75	98.46	99.09
chb19	100.00	100.00	100.00	100.00	100.00	100.00
chb20	99.71	99.88	99.54	100.00	100.00	100.00
chb21	81.83	80.97	82.74	93.89	89.20	98.37
chb22	95.72	97.02	94.49	95.00	92.50	97.50
chb23	94.93	94.48	95.38	97.05	96.60	97.44
Average	92.74	93.90	91.82	96.99	96.48	97.46

**Table 6:** The performance of 1 s SWT segments under proposed model using ZJU4H dataset.

	pa01	pa02	pa03	pa04	pa05	pa06	pa07	pa08	Average
**Accuracy**	92.31	84.73	99.72	91.56	99.44	99.33	86.94	100.00	94.25
**Sensitivity**	90.54	97.26	99.43	94.44	100.00	99.33	97.73	100.00	97.76
**Specificity**	96.27	82.73	100.00	88.68	98.91	99.33	86.63	100.00	94.07

**Table 7:** Comparison of recent segment-based seizure prediction studies conducted using CHB-MIT dataset with proposed work.

Reference	Dataset/No. of Patients	Feature/Preprocessing	Classifier	ACC	SEN	SPE
Khan et al. (2017) [19]	CHB-MIT/15	DWT	CNN	-	87.8	85.8
Truong et al. (2018) [17]	CHB-MIT/13	STFT	CNN	-	81.2	84.0
Acharya et al. (2018) [43]	Freiburg/5	Z-score normalization	CNN	88.7	90.0	95.0
Usman et al. (2021) [44]	CHB-MIT/22	CNN	LSTM	-	93.0	92.5
Ozcan et al. (2019) [45]	CHB-MIT/16	spectral band power, statistical moment, Hjorth parameters	3D CNN	-	85.7	-
Zhang et al. (2019) [42]	CHB-MIT/23	CSP	CNN	90.0	92.2	-
Gao et al. (2022) [46]	CHB-MIT/16	CNN	CNN	-	93.3	-
Usman et al. (2020) [47]	CHB-MIT/23	STFT	CNN-SVM	-	92.7	90.8
Abdelhameed et al. (2021) [48]	CHB-MIT/12	Z-score normalization	SCVAE	-	94.5	-
Zhang et al. (2021) [49]	CHB-MIT/13	M-SampEn	BiLSTM	80.1	86.7	74.1
Peng et al. (2021) [50]	CHB-MIT/17	FNN, SPR	CNN	-	85.4	-
**Proposed Method**	**CHB-MIT/23**	**SWT**	**CNN**	**96.99**	**96.48**	**97.46**

ACC, accuracy (%); SEN, sensitivity (%); SPE, specificity (%).

## Data Availability

The ZJU4H dataset is not publicly available due to privacy or ethical restrictions.
